# The mediating role of wellbeing in the relationship between health behaviors and quality of life in people with hepatogenous diabetes

**DOI:** 10.3389/fpubh.2025.1529158

**Published:** 2025-05-30

**Authors:** Ying Liang, Shuangqi Li, Lianxin Meng, Xisheng Wang, Zhichuan He, Qingmei Lu, Li Huang, Yijuan Li, Weiping Xie, Hongyun Ji

**Affiliations:** ^1^School of Nursing, Youjiang Medical University for Nationalities, Baise, Guangxi, China; ^2^Department of Gastroenterology, Affiliated Hospital of Youjiang Medical University for Nationalities, Baise, Guangxi, China; ^3^Guangxi Clinical Medical Research Center for Hepatobiliary Diseases, Baise, China; ^4^First Clinical Teaching Group, Baise Minzu Health School, Baise, Guangxi, China

**Keywords:** hepatogenous diabetes, wellbeing index, health behavior, quality of life, mediating effect

## Abstract

**Aim:**

This study aimed to investigate the relationship between health behaviors, wellbeing, and health-related quality of life (HRQoL) in patients with hepatogenous diabetes (HD), with a focus on exploring the mediating effect of the wellbeing index.

**Design:**

A cross-sectional analysis was conducted on 161 people with HD from January to December 2021 at a Grade III, Class A hospital's Department of Gastroenterology in Guangxi Province.

**Methods:**

Health behaviors were assessed using the Health-Promoting Lifestyle Profile (HPLP-C), HRQoL was measured with the Chronic Liver Disease Questionnaire (CLDQ), and emotional wellbeing was evaluated using the WHO-5 Wellbeing Index. Correlation analysis and a mediation model were used to explore the relationships between health behaviors, wellbeing, and HRQoL.

**Results:**

The results revealed a significant positive correlation between health behaviors and HRQoL (*r* = 0.385, *p* < 0.01) as well as between the wellbeing index and HRQoL (*r* = 0.513, *p* < 0.01). The wellbeing index partially mediated the relationship between health behaviors and HRQoL, accounting for 44.47% of the total effect. Despite generally favorable health behaviors, people with HD exhibited poor HRQoL (59.03 ± 17.47), with 28.6% experiencing depressive symptoms.

**Conclusion:**

Improving psychological wellbeing plays a crucial role in enhancing HRQoL among people with HD. Interventions should target both health behaviors and mental health to foster a more positive psychological state and improve overall patient outcomes.

## 1 Introduction

Hepatogenous diabetes (HD) is a metabolic disorder secondary to liver parenchymal damage, most seen as a late-stage complication of liver cirrhosis (LC), and is primarily characterized by hyperglycemia and impaired glucose tolerance (1, 2). Affecting 20–60% of cirrhotic patients (3), HD often develops outside of endocrinology departments, where clinical focus is on managing liver disease rather than blood glucose management (4). This oversight increases mortality rates in LC patients with HD and exacerbates complications like hepatic encephalopathy (5, 6), gastrointestinal bleeding (7), ascites, spontaneous bacterial peritonitis, and infections, and increasing the risk of progression to hepatocellular carcinoma (8). These compounded risks result in dramatically reduced 5-year survival rates for patients with LC and liver cancer (9). Additionally, managing HD is more challenging than managing type 2 diabetes mellitus (T2DM) due to complex treatment requirements and poorer prognosis. Patients often need prolonged pharmacological intervention and careful glucose management, resulting in substantial economic and psychological burdens, including depression and anxiety, which diminish their health-related quality of life (HRQoL) (10). Given the prevalence and impact of HD, prioritizing its management alongside liver disease is essential to improve patient survival and HRQoL in this vulnerable population.

Health behaviors and emotional states are important factors influencing HRQoL in chronic disease patients (11). Positive health behaviors, including regular physical activity, healthy eating habits, and effective stress management, are associated with improved physical and mental wellbeing in diabetes patients (12). For example, studies (13, 14) found that adopting a healthy diet and engaging in regular exercise significantly improved HRQoL in diabetes patients. This supports the notion that health behaviors can mitigate some of the physiological and psychological burdens of chronic diseases like diabetes. Similarly, emotional states, such as anxiety and depression, play a critical role in determining HRQoL. Negative emotional states, such as those caused by the psychological stress of managing a chronic illness, have been shown to lower HRQoL in liver cirrhosis patients. Research (15, 16) demonstrated a significant negative correlation between emotional distress and HRQoL in patients with hepatitis B cirrhosis, suggesting that psychological wellbeing is a crucial component in the overall health of these patients.

In understanding the behavioral and psychological dimensions of chronic illness, the Health Belief Model (HBM) offers a useful theoretical framework. The HBM suggests that individuals' health behaviors are influenced by their perceived susceptibility to illness, perceived severity of the condition, perceived benefits and barriers to taking action, and cues to action (17, 18). Importantly, emotional states such as fear, anxiety, or depression can modulate these perceptions, thereby influencing health behaviors. For example, people with low psychological wellbeing may underestimate the benefits of adherence to diet or medication, or feel overwhelmed by barriers to self-care.

Extending this model, wellbeing—characterized by positive mood, stress regulation, and psychological resilience—may not only influence health behaviors but also serve as a mediator in the pathway between health behaviors and HRQoL. That is, better emotional wellbeing may enhance engagement in healthy behaviors, which in turn improve perceived and actual HRQoL. Conversely, poor emotional wellbeing may inhibit health behaviors, thereby negatively impacting HRQoL. In this sense, emotional wellbeing and health behaviors may have reciprocal and mediating effects on one another in relation to HRQoL. Moreover, since HRQoL encompasses both physical and psychological domains, the role of emotional wellbeing is central to understanding how individuals experience and manage chronic conditions. Thus, incorporating emotional wellbeing into the HBM framework allows for a more nuanced understanding of how psychological factors influence behavior and ultimately affect HRQoL outcomes. This integrated perspective suggests that interventions targeting emotional wellbeing may not only improve mental health directly but also indirectly enhance HRQoL by promoting healthier behaviors.

Despite the recognition of the individual effects of health behaviors and psychological wellbeing on HRQoL, there remains a significant gap in understanding how these factors interact to influence HRQoL among people with HD. Specifically, it is unclear whether psychological wellbeing mediates the relationship between health behaviors and HRQoL in this population. People with HD face the dual burden of liver disease and diabetes, potentially experiencing more severe physical symptoms and psychological challenges than patients with a single chronic condition. Understanding this interplay is critical because it may reveal pathways through which interventions can be most effective. To our knowledge, no studies have specifically examined the mediating role of psychological wellbeing in the relationship between health behaviors and HRQoL among people with HD. Investigating this mediating effect is novel and significant, as it could provide insights into targeted intervention strategies that simultaneously address physical health behaviors and psychological wellbeing to enhance HRQoL of people with HD.

This study aims to assess the levels of health behaviors, psychological wellbeing, and HRQoL among people with HD while examining the relationships between these factors and investigating the mediating role of psychological wellbeing in the relationship between health behaviors and HRQoL. We propose the following hypotheses: H1 posits that positive health behaviors are significantly associated with higher HRQoL in people with HD; H2 suggests that greater psychological wellbeing correlates with higher HRQoL; and H3 asserts that psychological wellbeing mediates the relationship between health behaviors and HRQoL, indicating that positive health behaviors enhance psychological wellbeing, which subsequently leads to improved HRQoL. By exploring these interconnections, this study seeks to address a critical gap in the existing literature and establish a theoretical foundation for developing interventions aimed at enhancing HRQoL in people with HD. Furthermore, understanding the mediating role of psychological wellbeing can inform comprehensive care strategies that promote positive health behaviors while addressing psychological needs, ultimately leading to improved patient outcomes.

## 2 Methods

### 2.1 Study design

This study employs a cross-sectional design to examine the relationships between health behaviors, psychological wellbeing, and HRQoL among patients diagnosed with HD. A cross-sectional design was chosen due to its efficiency in assessing multiple variables at a single point in time, making it suitable for exploring associations and potential mediating effects within the study population.

Participants were selected using a convenience sampling method between January and December 2021 at a tertiary Grade A hospital in Baise, Guangxi Province, China. This hospital was chosen due to its status as a major healthcare provider in the region, with a high volume of patients suffering from liver disease, including HD. It is a critical medical hub at the intersection of Guangxi, Yunnan, and Guizhou provinces, making it an ideal location to recruit a representative sample of people with HD. Serving a large catchment area, the hospital draws patients from diverse socioeconomic backgrounds, rural and urban settings, and different ethnic groups, reflecting the broader demographic characteristics of people with HD in southern China. Furthermore, the hospital's multidisciplinary approach to treating liver disease creates an environment where patients with dual conditions like HD receive specialized care. This allows the study to capture data on patients whose health behaviors and psychological wellbeing are managed in a holistic, comprehensive healthcare system, which mirrors the broader care patterns observed in specialized HD treatment settings across the region.

Strict data collection and quality control procedures were conducted to ensure data accuracy and reliability, as detailed in Section 2.4. These measures included comprehensive training for the research team, rigorous data validation, and adherence to ethical standards, which helped minimize bias and ensure the consistency of the data collected.

### 2.2 Participants

The study was approved by the Ethics Committee of the Youjiang Medical University for Nationalities with the Ethical Approval Number: 2020120601. All patients provided written informed consent. During the study period, a total of 161 questionnaires were distributed to patients who met these criteria. Questionnaires were excluded from the analysis due to incomplete data, specifically when responses were missing more than 10% of the total items. This criterion particularly applied to critical items essential for the analysis, including demographic information and key clinical variables. The collection was done within 1 week of hospital admission to ensure that the patients' condition was stable and that their responses reflected their typical health status.

To ensure the relevance and consistency of the data collected, strict inclusion and exclusion criteria were applied. For inclusion criteria, participants were required to (1) have a confirmed diagnosis of liver cirrhosis based on the 2019 revised Chinese guidelines for the management of liver cirrhosis (19); (2) meet the diagnostic criteria for diabetes mellitus as defined in the 2020 edition of China's Guidelines for the Prevention and Control of Type 2 Diabetes Mellitus (20), including a fasting blood glucose (FBG) level of ≥7.0 mmol/L, a 2-h postprandial glucose (2hPG) level of ≥11.1 mmol/L, or a glycated hemoglobin (HbA1c) level of ≥6.5%; (3) have liver cirrhosis diagnosed concurrently with or prior to diabetes; (4) lack a personal or familial history of diabetes prior to the liver disease diagnosis; (5) be aged between 30 and 70; and (6) possess the cognitive and behavioral capacity to give informed consent and participate in the study.

Patients were excluded from the study (1) if they were currently or had previously been treated with corticosteroids, (2) had any psychiatric or cognitive impairments, (3) or suffered from severe complications such as gastrointestinal bleeding, hepatic encephalopathy, or liver cancer. (4) Furthermore, individuals with other significant health issues, including malignant tumors or severe cardiac or renal diseases, were also excluded to ensure that the study focused specifically on the impact of HD on HRQoL without confounding health conditions.

### 2.3 Measures

#### 2.3.1 Permission to reuse and copyright

Demographic information and clinical data were meticulously extracted from electronic health records (EHRs), ensuring high accuracy and reliability of the data. For patients diagnosed with HD, we collected a broad spectrum of demographic details, including gender, education level, income status, age, healthcare payment method, place of residence, and occupational status. These variables are crucial as they can influence disease management outcomes and access to healthcare resources, which are integral to understanding disparities in health behaviors and HRQoL among people with HD.

Clinical data gathered encompassed days of hospitalization, the duration and etiology of cirrhosis, as well as patients' tobacco and alcohol history. Additionally, precise anthropometric measurements (height, weight), blood glucose values, glycosylated hemoglobin levels, and liver function parameters were recorded. These measures were taken using standardized protocols to ensure consistency across the study.

This comprehensive approach to data collection was designed to facilitate a detailed understanding of the participants' backgrounds and their disease profiles, thereby enriching the analysis of how wellbeing indices, health behaviors, and HRQoL interact in the context of HD.

#### 2.3.2 Chronic liver disease questionnaire

To assess health-related quality of life (HRQoL) in this study, we employed the Chronic Liver Disease Questionnaire (CLDQ), a disease-specific instrument developed by Younossi et al. in 1999 to capture the symptom burden and daily functional impairment experienced by patients with chronic liver disease and cirrhosis (21). We used the Chinese-translated and validated version by Wu et al. (22), which has demonstrated robust reliability and construct validity within Chinese populations with liver disease.

The CLDQ consists of 29 items categorized into six dimensions: abdominal symptoms (SA), fatigue (FA), systemic symptoms (SS), activity (AC), emotional function (EF), and worry (WO). These dimensions are crucial for a comprehensive understanding of the diverse impact of liver disease on patients' daily lives. Each item is rated on a seven-point scale, from “never” (score of 1) to “always” (score of 7), offering a detailed assessment of the severity and frequency of symptoms and concerns. The total score is the sum of the scores across all dimensions, which is standardized to a full score of 100, with higher scores indicating better QoL. The reliability of the CLDQ in this study was demonstrated by Cronbach's alpha coefficients for each dimension: AS (0.84), FA (0.90), SS (0.75), AC (0.76), EF (0.84), and WO (0.85), which indicate good to excellent internal consistency. These values underscore the robustness of the CLDQ in measuring QoL among patients with HD, particularly within the Chinese population. Previous studies using the CLDQ have consistently demonstrated strong construct validity, and its ability to capture QoL outcomes in various liver diseases suggests it is appropriate for use with people with HD (22).

Our target population was composed exclusively of patients with HD, defined as type 2 diabetes occurring in the context of pre-existing or concurrent liver cirrhosis, in the absence of a personal or family history of diabetes. Given this clinical background, liver dysfunction and cirrhosis-related complications represent the primary etiology and driver of metabolic and systemic symptoms in this population, rather than traditional insulin resistance or metabolic syndrome.

CLDQ was selected over generic HRQoL instruments (e.g., SF-36 or EQ-5D) or diabetes-specific tools (e.g., DQOL or ADDQoL) because it is specifically tailored to capture the broad spectrum of symptoms, emotional burdens, and activity limitations associated with liver disease and its complications, such as fatigue, abdominal discomfort, and hepatic-related worry. Since our study focused on the impact of HD, essentially a liver-disease–driven metabolic condition on quality of life, CLDQ offered superior content validity and clinical relevance.

#### 2.3.3 Health promoting lifestyle profile-Chinese

The Health Promoting Lifestyle Profile-Chinese (HPLP-C) adopted in this research is an translated version of the original scale developed by Cao et al. (23), ensuring its relevance and applicability in measuring lifestyle behaviors that contribute to health promotion among Chinese populations.

The HPLP-C comprises 40 items across six dimensions: nutrition, health responsibility, life appreciation, social support, physical activity, and stress management. Responses to each item are collected using a Likert 4-point scale, ranging from 1 (“never”) to 4 (“routinely”), where higher scores indicate healthier lifestyle. The total score for the scale ranges from 40 to 160, where scores between 100 and 160 indicate a healthy lifestyle characterized by effective health-promoting behaviors. In contrast, scores between 40 and 99 suggest an unhealthy lifestyle, highlighting areas where interventions may be needed (17).

The validity of the HPLP-C in chronic disease populations has been well established in previous studies (24, 25). While the tool has not been specifically validated in the HD population, its wide use in similar contexts, particularly among Chinese patients with chronic diseases, supports its use in this study. In addition, the conceptual framework of health promotion behaviors that the HPLP-C assesses, such as nutrition, physical activity, and stress management, is highly relevant to the lifestyle factors influencing HRQoL in people with HD, making it appropriate for exploring these relationships.

The reliability of the HPLP-C is excellent, with a Cronbach's alpha coefficient of 0.93, indicating high internal consistency across the scale. This level of reliability ensures that the HPLP-C provides consistent and dependable measurements when assessing health behaviors in this population. Given its high reliability and relevance to health-promoting behaviors in chronic disease management, the HPLP-C is a robust tool for both research and clinical assessments within Chinese populations, including people with HD.

#### 2.3.4 World Health Organization-5 wellbeing index

The World Health Organization-5 wellbeing index (WHO-5) was proposed by Bech.P and then revised through the WHO Collaborating Center for Psychological Research (26). The WHO-5 has been effectively used in various clinical settings and among different populations, including patients with chronic illnesses. The scale is a subjective feeling scale, which was used as an important psychological parameter to measure the level of mental health of individuals. It contains a total of five entries, each involving six levels, “at no time”, “some of the time”, “less than half the time”, “more than half the time”, “most of the time”, and “all of the time”, corresponding to a score of 0–5 (26). Raw score was multiplied by four to get a total score ranging from 0 to 100. A higher score on this scale indicates a more positive mental state and a better overall HRQoL. A score below 50 indicates poor wellbeing and suggests the need for further investigation into potential symptoms of depression, while a score of 28 or below is indicative of depression (27).

The reliability of the WHO-5 in this study is demonstrated by its Cronbach's alpha coefficient of 0.86 (28), indicating good internal consistency. This level of reliability ensures that the WHO-5 is a dependable measure for assessing wellbeing among people with HD. While the WHO-5 has not been specifically validated in people with HD, its broad use in chronic illness populations, including those with liver diseases and diabetes—supports its application in this context. In addition to its reliability, WHO-5 has demonstrated strong validity in various health settings, showing consistent correlations with other psychological and HRQoL measures. Given the psychological burden associated with chronic diseases such as HD, the WHO-5 is an appropriate tool for evaluating mental health and its impact on HRQoL in this population.

To address potential concerns about conceptual overlap between the WHO-5 and the Emotional EF subscale of the CLDQ, we examined the theoretical and operational distinctions between the two measures. The WHO-5 is a positively worded, general wellbeing index designed to assess core aspects of subjective psychological wellbeing, such as mood, vitality, and interest in life, over the past 2 weeks. In contrast, the EF subscale of the CLDQ is a disease-specific measure that evaluates emotional responses (e.g., irritability, frustration, and worry) directly related to chronic liver disease and its complications. While both scales tap into affective states, the WHO-5 reflects a broader and more general construct of mental wellbeing, whereas the CLDQ-EF is anchored in the context of liver disease–related emotional burden. Additionally, the instruments differ in item content, scoring approach, and theoretical orientation: the WHO-5 aligns with positive psychology and screening for depression, while the CLDQ-EF focuses on negative affect specific to liver disease. This conceptual and methodological distinction supports the use of the WHO-5 as an independent mediator in the analysis.

### 2.4 Data collection and quality control procedures

(1) Prior to the commencement of the survey, extensive training was conducted for all research team members. These training covered the survey's objectives, the methodologies employed, and the correct procedures for administering the questionnaire, ensuring uniformity and reliability in data collection across all team members.
(2) At the point of hospitalization and once a stable medical condition was confirmed, eligible patients were thoroughly introduced about the study's purpose and its potential implications. Detailed explanations were provided to secure informed consent, ensuring that all participants were willingly and fully informed before participating in the study.
(3) Patients using smartphones were presented with a QR code, which they scanned to access and immediately submit their responses to the questionnaire. For patients without smartphone access, the questionnaire was distributed and completed face-to-face, with responses collected on the spot. Detailed instructions on how to complete the questionnaire, along with necessary precautions, were clearly communicated to all respondents to ensure accurate and consistent data collection.
(4) Laboratory results were systematically retrieved from the patient medical records. For those patients with multiple lab results recorded within the survey period, the results closest to the survey date were chosen. This approach was adopted to ensure that the most relevant and current data were used for analysis.
(5) Each questionnaire, both electronic and paper-based, was meticulously checked for completeness and correctness before being officially submitted or collected. All collected data underwent a double-entry process, particularly the data from paper-based questionnaires. This was performed blind, where the data entry personnel were not aware of the study hypotheses or the health status of participants, particularly for paper-based questionnaires. Such blinding was crucial to minimize any potential for subjective bias or data entry errors, thus enhancing the reliability and validity of the data collected.
(6) Throughout the data collection process, strict adherence to ethical standards was maintained. All patient data was treated with the highest confidentiality, and all interactions were conducted with respect for the patients' privacy and dignity. Ethical guidelines were rigorously followed to ensure compliance with both local and international standards, safeguarding participant welfare and data integrity.

### 2.5 Data analysis

Statistical analysis was conducted using SPSS 26.0. Descriptive analysis was performed to summarize general demographic information, clinical characteristics, and the HRQoL of patients with HD. The study included a total of 161 patients, with a sample size of 93 participants calculated using PASS 15.0 to achieve 95% power to detect a medium effect size (*f*^2^ = 0.15) in the context of a regression model. This setup ensures sufficient statistical power to detect an effect of the independent variable on the outcome, assuming the effect size remains at or above 0.15.

Normally distributed continuous variables, such as age and laboratory measurements (blood glucose levels, glycosylated hemoglobin levels), were presented as mean ± standard deviation (*x* ± sd). Categorical variables, including gender, education level, and healthcare payment method, were expressed as frequency (percentage). Correlation analysis was used to analyze the correlation between the healthy behaviors and wellbeing index of people with HD with the HRQoL.

To assess the mediating role of psychological wellbeing in the relationship between health behaviors and HRQoL, we employed a bootstrapping method using PROCESS macro for SPSS (29). Bootstrapping is a non-parametric resampling technique that generates confidence intervals for the indirect effects, providing a more rigorous test of mediation than traditional stepwise regression. A 5,000-sample bootstrap was used to estimate the indirect effects of psychological wellbeing, with bias-corrected confidence intervals (CI) calculated at the 95% level. The mediation analysis involved three key steps: Step 1 explored the direct impact of health behaviors on the dependent variable, HRQoL. Step 2 assessed the relationship between independent variable, health behaviors, and the mediating variable, psychological wellbeing, to establish a significant association. Step 3 integrated both the independent variable and the mediating variable, examining how much of the effect of health behaviors on HRQoL was mediated through psychological wellbeing. The mediation effect was considered statistically significant if the 95% CI for the indirect effect did not include zero. If the inclusion of psychological wellbeing reduced the direct effect of health behaviors on HRQoL, partial mediation was inferred; if the direct effect became non-significant, full mediation was indicated. A *p*-value < 0.05 was set for statistical significance across all tests. This comprehensive modeling quantified the extent to which psychological wellbeing could mediate the effects of health behaviors on HRQoL, providing valuable insights into potential therapeutic targets for enhancing patient outcomes in HD management.

## 3 Results

### 3.1 Demographic and clinical characteristics of people with HD

A total of 161 people with HD were enrolled in this study, with a mean age of ~54.97 ± 10.65 years. Most patients were older adults, with 64.6% being above the age of 50 years. The cohort also displayed significant variability in education levels and income, with most patients having lower education and income levels. Notably, a substantial proportion of patients were smokers (43.5%) and consumed alcohol (61.5%). Clinically, the patients exhibited elevated mean FBG of 8.74 ± 4.57 mmol/L and HbA1c levels of 7.65 ± 1.85%, indicating poor glycemic control, along with altered lipid profiles. Detailed information of demographic and clinical characteristics is shown in Table 1.

**Table 1:** Characteristics of patients.

**Characteristic**	***n***	**%**	**Mean ±SD**
**Demographic characteristics**			
Age, years			54.97 ± 10.65
≤ 30	3	1.9	
30–40	10	6.2	
41–50	44	27.3	
51–60	47	29.2	
≥61	57	35.4	
**Gender**			
Men	132	82.0	
Women	29	18.0	
**Educational level**			
Middle school and below	113	70.2	
High school	24	14.9	
University or above	24	14.9	
**Personal monthly average income**			
≤ 3,000	108	67.1	
>3,000	53	32.9	
**Smoking**			
Yes	70	43.5	
No	91	56.5	
**Alcohol**			
Yes	99	61.5	
No	62	38.5	
**Disease characteristics**			
Fasting blood glucose (FBG), mmol/L			8.74 ± 4.57
HbA1c, %			7.65 ± 1.85
Total cholesterol (TC), mmol/L			3.66 ± 1.58
Triglyceride (TG), mmol/L			2.01 ± 5.46

### 3.2 Assessment of HRQoL, health behavior, and wellbeing index

The HRQoL among patients with HD, assessed by using the CLDQ, revealed a moderate overall score of 59.03 ± 17.47. Emotional functioning achieved the highest score (16.68 ± 5.46), suggesting a relatively favorable emotional wellbeing among participants. Conversely, lower scores in the activity (6.29 ± 2.58) and abdominal symptoms (6.67 ± 2.53) domains indicate that these aspects are more significantly impacted by HD. In terms of health behaviors, the HPLP-C scores reflected varied engagement among patients. Participants reported high scores in stress management (27.94 ± 5.55) and life appreciation (25.79 ± 4.97), while physical activity scores were considerably lower (8.08 ± 2.67), indicating an area that may require further encouragement and support. Furthermore, the WHO-5 wellbeing index revealed a wide range of emotional wellbeing with an average score of 50.58 ± 27.15. Specifically, 50.3% of patients reported feeling happy, 21.1% showed diminished happiness, and 28.6% exhibited symptoms indicative of depression. This distribution underscores the varying levels of emotional wellbeing within the cohort, with a significant proportion of patients potentially at risk for mental health challenges. Detailed scores of each questionnaire are listed in Table 2.

**Table 2:** Scores of CLDQ, HPLP-C, and WHO-5 among people with HD.

**Scale**	**Dimension**	**Mean ±SD**
CLQD	Abdominal symptoms	6.67 ± 2.53
	Fatigue	8.80 ± 4.25
	Systemic symptoms	11.99 ± 2.83
	Activity	6.29 ± 2.58
	Emotional function	16.68 ± 5.46
	Worry	8.61 ± 4.22
	Total	59.03 ± 17.47
HPLP-C	Nutrition	15.45 ± 3.05
	Health responsibility	18.83 ± 4.96
	Life appreciation	25.79 ± 4.97
	Social support	20.62 ± 3.67
	Physical activity	8.08 ± 2.67
	Stress management	27.94 ± 5.55
	Total	113.70 ± 17.41
WHO-5 wellbeing index	Happy (≥50)	74.47 ± 12.88
	Diminished happiness (29–50)	38.00 ± 5.50
	Depressed ( ≤ 28)	17.83 ± 7.81
	Total	50.58 ± 27.15

### 3.3 Relationships between HRQoL, health behaviors and wellbeing index

The analysis revealed that HRQoL was positively correlated with overall health behaviors (*r* = 0.385, *p* < 0.01), indicating that improvements in health behaviors are associated with better perceived HRQoL among people with HD. Among specific health behaviors, nutrition (*r* = 0.389, *p* < 0.01), life appreciation (*r* = 0.360, *p* < 0.01), and stress management (*r* = 0.302, *p* < 0.01) showed particularly moderate correlations with HRQoL, suggesting that these behaviors play a significant role in improving patients' overall HRQoL.

There was also a significant positive correlation between HRQoL and the wellbeing index (*r* = 0.513, *p* < 0.01). This indicates that higher levels of wellbeing are linked to better HRQoL, emphasizing the importance of psychological health in overall life satisfaction.

Additionally, health behaviors were positively correlated with the wellbeing index (*r* = 0.401, *p* < 0.01), with specific behaviors like stress management (*r* = 0.368, *p* < 0.01) and life appreciation (*r* = 0.403, *p* < 0.01) showing moderate associations. This highlights that engaging in positive health behaviors not only supports physical health but also plays a critical role in enhancing psychological wellbeing. A full summary of the coefficients of correlation can be found in Table 3.

**Table 3:** Correlations among HRQoL, health behaviors and wellbeing index.

**Variables**	**1**	**2**	**3**	**4**	**5**	**6**	**7**	**8**	**9**
1.Quality of life	1								
2.Health behaviors	0.385^**^	1							
3.Nutrition	0.389^**^	0.705^**^	1						
4.Health responsibility	0.197^*^	0.794^**^	0.466^**^	1					
5.Life appreciation	0.360^**^	0.774^**^	0.436^**^	0.444^**^	1				
6.Social support	0.130	0.571^**^	0.394^**^	0.300^**^	0.437^**^	1			
7.Physical activity	0.295^**^	0.636^**^	0.390^**^	0.539^**^	0.364^**^	0.167^*^	1		
8.Stress management	0.302^**^	0.761^**^	0.483^**^	0.573^**^	0.478^**^	0.187^*^	0.436^**^	1	
9.Wellbeing index	0.513^**^	0.401^**^	0.299^**^	0.135	0.403^**^	0.268^**^	0.227^**^	0.368^**^	1

^*^p < 0.05.

^**^p < 0.01.

Furthermore, to assess whether there was undue conceptual overlap between the wellbeing index and the more emotionally focused dimensions of the CLDQ, we examined the correlations between WHO-5 scores and the CLDQ emotional (*r* = 0.585, *p* < 0.01) and worry (*r* = 0.475, *p* < 0.01) subscales. Both correlations were moderate, indicating that while these dimensions are related, they are not redundant. These findings support the notion that WHO-5 measures a broad sense of positive psychological wellbeing, whereas the CLDQ subscales capture disease-specific emotional concerns. A full summary of the coefficients of correlation can be found in Table 3.

### 3.4 Mediating effect of wellbeing index between health behaviors and HRQoL among people with HD

Based on the Health Belief Model (HBM), it is hypothesized that the wellbeing index mediates the relationship between health behaviors and HRQoL in patients with HD. The HBM suggests that individuals' health-related behaviors are influenced by their perceptions of health risks and benefits, which in turn affect their overall wellbeing and HRQoL (30).

**Figure 1:**
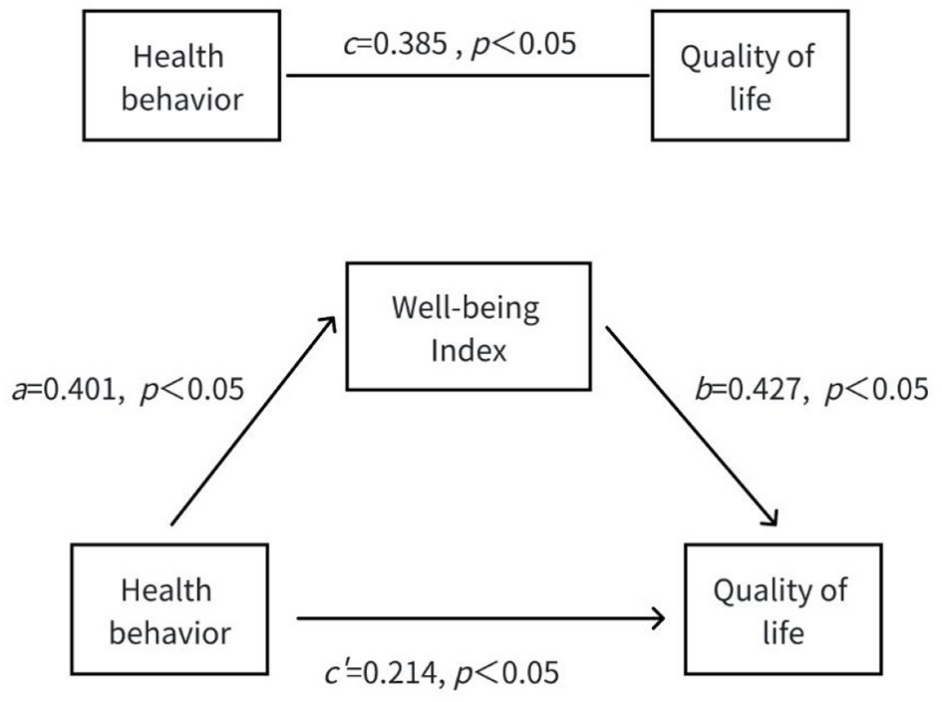
Mediating role of people with HD' wellbeing index between health behaviors and HRQoL. c is total effect path, a*b is direct effect path, c′ is indirect effect path. ^*^p < 0.05.

To test this hypothesis, a regression analysis was conducted using the entry method to determine whether the wellbeing index mediates the effect of health behaviors on HRQoL. The health behavior score of people with HD was used as the independent variable, and the HRQoL score was used as the dependent variable. The detailed results are presented in Table 4. The analysis followed a standard three-step mediation model: In the first step, health behaviors were significantly associated with HRQoL (*R*^2^ = 0.148, *p* < 0.001), indicating that better health behaviors were linked to higher HRQoL among people with HD. In the second step, health behaviors were significantly associated with the wellbeing index (*R*^2^ = 0.160, *p* < 0.001), suggesting that positive health behaviors are related to higher wellbeing. In the third step, when the wellbeing index was included in the model, it was significantly associated with HRQoL (*R*^2^ = 0.301, *p* < 0.001). Meanwhile, the effect of health behaviors on HRQoL weakened from β = 0.385 to β = 0.214, but it remained significant (*p* < 0.05). This indicates that the wellbeing index partially mediates the relationship between health behaviors and HRQoL. The contribution of the mediating effect to the total effect was calculated as (0.401 × 0.427)/0.385 = 44.47%. Thus, the wellbeing index mediates 44.47% of the total effect of health behavior on HRQoL. The specific mediation model is illustrated in Figure 1.

**Table 4:** Mediating effect analysis of wellbeing index.

**Model**	**Step 1 HRQoL**				**Step 2 wellbeing index**				**Step 3 HRQoL**			
	***B***	***SD***	**β**	***t***	***B***	***SD***	**β**	***t***	***B***	***SD***	**β**	***t***
Health behavior	0.386	0.073	0.385	5.262^***^	0.625	0.113	0.401	5.513^***^	0.215	0.073	0.214	2.950^**^
Wellbeing index									0.275	0.047	0.427	5.881^***^
*F*	27.685^***^				30.388^***^				34.057^***^			
*R* ^2^	0.148				0.160				0.301			

^**^p < 0.01.

^***^p < 0.001.

## 4 Discussion

This study aimed to investigate the relationship between health behaviors, wellbeing, and HRQoL in people with HD, while also exploring the mediating role of the wellbeing index. The results showed that HRQoL was generally poor (59.03 ± 17.47), despite relatively favorable health behaviors (114.45 ± 18.36). The wellbeing index revealed that only 50.3% of patients felt happy, while 28.6% experienced depressive symptoms. Importantly, better health behaviors (*r* = 0.385, *p* < 0.01) and higher wellbeing (*r* = 0.513, *p* < 0.01) were both significantly associated with improved HRQoL. Furthermore, the wellbeing index partially mediated the relationship between health behaviors and HRQoL, accounting for 44.47% of the total effect, highlighting the crucial role of psychological wellbeing in enhancing overall HRQoL in people with HD.

### 4.1 Comparisons with other patient populations

The study shows that the HRQoL score for people with HD was significantly lower than the national norm of multi-center study patients (75.1 ± 15.0) (31), highlighting a critical area for clinical attention. The poor HRQoL in people with HD can be attributed to factors such as recurring illness, long disease duration, and complications like ascites and infections, which severely affect both their physical and mental health. Symptoms such as fatigue, weakness, and itching further contribute to the reduced HRQoL, placing people with HD well below the general population. Despite this, people with HD demonstrated generally good health behaviors, reflecting improved health literacy likely due to advancements in medical practices, healthcare development, and the widespread promotion of health education. However, this finding differs from previous research (23) on incarcerated individuals with chronic diseases, suggesting that variations in population, setting, and timing may account for the differences. Additionally, the wellbeing index score for people with HD was lower than that of patients with T2DM (70.67 ± 16.98) (32) and those undergoing maintenance hemodialysis (55.58 ± 16.47) (33), indicating widespread negative emotions in this population. Research (25) suggests that the complex pathogenesis of cirrhosis and HD, combined with multiple symptoms, challenging treatments, and poor prognosis, contributes to the high psychological burden. Long-term medication and dietary restrictions further add to the economic and psychological pressures, making people with HD more prone to depression and anxiety, which in turn negatively impact their HRQoL.

### 4.2 Role of health behaviors and wellbeing index in enhancing HRQoL

This dual impact of health behaviors on HRQoL highlights the importance of both physical and psychological aspects. On one hand, improved health behaviors, such as better stress management and life appreciation, directly enhance HRQoL. On the other hand, health behaviors also improve the wellbeing index, which in turn positively affects HRQoL. The significant positive correlation between the wellbeing index and HRQoL (*r* = 0.513, *p* < 0.01) demonstrates that patients with a more positive psychological state tend to have better HRQoL, and health behaviors play a key role in fostering such a positive mindset. The wellbeing index, which is used as a screening tool for depression in people with HD, further underscores this relationship. Previous research (34) showed that over 50% of cirrhosis patients experience negative emotions such as anxiety and depression, higher than the rates for hypertension patients (15%) and COPD patients (30%). Such negative emotions can diminish self-worth, may impair adherence to treatment and reduce prognosis and HRQoL (28).

### 4.3 Clinical implications and recommended interventions

The study's findings indicate that improved health behaviors significantly enhance psychological wellbeing, which in turn positively influences the HRQoL among people with HD. Regression analyses across three steps validated this mediating mechanism. Specifically, health behaviors were significantly associated with HRQoL (β = 0.385, *t* = 5.262, *p* < 0.001) and wellbeing (β = 0.401, *t* = 5.513, *p* < 0.001). When both predictors were included, the wellbeing index remained a strong independent predictor of HRQoL (β = 0.427, *t* = 5.881, *p* < 0.001), while the direct effect of health behavior on HRQoL was attenuated (β = 0.214, *t* = 2.950, *p* < 0.01), confirming partial mediation. The overall model explained 30.1% of the variance in HRQoL (*R*^2^ = 0.301), underscoring the clinical importance of addressing both behavioral and psychological factors in routine care. To interpret the effect size, the standardized coefficient of the wellbeing index (β = 0.427) suggests a moderate-to-large practical impact on HRQoL, according to Cohen's conventions. The *R*^2^ increment from 0.160 to 0.301 after including psychological wellbeing indicates substantial explanatory power gained by adding this mediator. This mediating relationship underscores the critical interplay between physical health practices and mental wellbeing in managing HD. Understanding this mechanism has profound implications for clinical practice, suggesting that comprehensive care strategies should integrate both behavioral and psychological interventions to optimize patient outcomes.

Our findings align with previous research demonstrate the positive impact of health behaviors on HRQoL in chronic disease populations. For instance, Tapehsari et al. reported that regular physical activity and healthy dietary habits significantly improved HRQoL in patients with type T2DM (35), similar to our observations in people with HD. Additionally, studies by Lee et al. (36) highlighted the role of psychological wellbeing as a mediator between self-management and HRQoL in in patients with hypertension, reinforcing the notion that mental health is integral to managing chronic illnesses. Targeted interventions should be implemented at multiple levels, including departmental, hospital management, and individual levels, to provide comprehensive support such as enhanced health education, psychological support, and individualized care plans.

At the Departmental Level: Clinical doctors should implement standardized screenings for OGTT and HbA1c when cirrhosis patients are admitted enabling early detection and timely treatment of HD. This would facilitate better control of both liver function and blood sugar levels. Given the prevalence of negative emotions among people with HD, medical staff should prioritize addressing their psychological wellbeing. Since people with HD often have compromised liver function, managing blood sugar should focus on improving liver health. For patients with hepatitis B-induced HD, antiviral treatments should be actively pursued. Furthermore, as many people with HD have lower educational backgrounds, nursing staff should provide personalized health education tailored to their specific needs.

At the Hospital Management Level: Hospitals should establish case management files and strengthen pre-discharge assessments, follow-up care, and continued care services. Promoting the “Internet + Nursing” service model is crucial to monitor the entire health cycle, not just the treatment phase. This approach can improve patient and family self-management abilities, leading to better maintenance of liver and blood sugar function and a reduction in complications.

At the Individual Level: Patients should actively seek out information about their disease through various channels, cultivate an optimistic mindset, and remain determined in managing their condition. By engaging in proactive self-management, patients can shift from passive treatment to taking an active role in their care, thus conserving healthcare resources, and improving their overall health outcomes.

Furthermore, the findings support the application of the HBM in designing educational strategies. By targeting patients' perceptions of disease severity, susceptibility, and self-efficacy, health education can effectively shift health behaviors and improve outcomes. For instance, patients who understand the consequences of poor glycemic control in HD are more likely to adhere to diet and medication, especially when educational programs address psychological barriers such as fear or fatalism. By reframing our findings through the lens of health education and promotion, we propose that future intervention programs should be theory-based, patient-centered, and emotionally responsive. This approach not only enhances the clinical relevance of the study but also solidifies its contribution to the field of health education research.

### 4.4 Limitations

This study confirmed the mediating effect of the wellbeing index between health behaviors and HRQoL in patients with HD. However, However, several limitations should be acknowledged. The cross-sectional design used in this study limits the ability to infer causal relationships between health behaviors, psychological wellbeing, and HRQoL. While associations were identified, future longitudinal studies are necessary to establish causality and explore how these relationships evolve over time. Potential confounders such as glycemic and lipid control were not adjusted, which may independently influence psychological wellbeing and HRQoL and thus confound the observed relationships. Future research should incorporate these variables in multivariate models for more precise analysis.

In addition, due to limitations in human resources and time, the study only included 161 patients from a tertiary hospital. Future research should aim to conduct multi-center, large-sample studies to provide more robust scientific evidence for improving the HRQoL of people with HD. The study was conducted during the COVID-19 pandemic, which may have affected healthcare access, treatment adherence, and lifestyle behaviors. While hemodialysis services were maintained at the study site, other aspects of diabetes management—such as dietary counseling and physical activity recommendations—may have been inconsistently delivered. Pandemic-related stress and reduced social support could also have influenced emotional wellbeing and health behaviors. Although these factors were not directly measured, future studies should consider pandemic-related variables or compare pre- and post-pandemic cohorts.

Finally, while we used established instruments (CLDQ, HPLP-C, WHO-5), these tools have not been specifically validated in patients with HD, which may affect the measurement accuracy of health behaviors, psychological wellbeing, and HRQoL in this population. Future research should address these limitations to provide more robust evidence.

## 5 Conclusion

This study shows that while people with HD demonstrate favorable health behaviors, their HRQoL is significantly reduced, with many experiencing depressive symptoms. The wellbeing index partially mediates the relationship between health behaviors and HRQoL, highlighting the importance of psychological wellbeing. To improve HRQoL in people with HD, interventions should target both physical and mental health, emphasizing mental health support alongside health behavior improvements. A comprehensive approach, including clinical screenings, personalized education, and self-management, is essential for better patient outcomes.

## Data Availability

The original contributions presented in the study are included in the article/supplementary material, further inquiries can be directed to the corresponding authors.
